# Choosing connection: relational values as a career choice motivation predict teachers’ relational goal setting

**DOI:** 10.3389/fpsyg.2023.1147276

**Published:** 2023-05-17

**Authors:** Lia Oberhauser, Silke Hertel

**Affiliations:** ^1^Lia Oberhauser, Heidelberg School of Education, Heidelberg University and University of Education Heidelberg, Heidelberg, Germany; ^2^Institute of Education Science, Heidelberg University, Heidelberg, Germany

**Keywords:** factors influencing teaching choice, teachers’ goal orientations, student-teacher relationship, educational interest, social utility values

## Abstract

Relational goals have a positive impact on teachers’ classroom performance, but little is known about the antecedents of these goals. One of the most important reasons for choosing teaching as a career is the desire to work with children/adolescents. This study examined this reason along with other relational career choice motives as predictors of relational goal orientation, complementing other studies that have examined the relationship between reasons for career choice and goal orientations but did not consider the relational component. We hypothesized that relational motives for career choice would predict relational goal setting for teaching better than other reasons for career choice. The sample comprised *N* = 167 student teachers at a large German university who answered an online questionnaire assessing motivations for choosing teaching as a career, professional self-concepts and relational goal orientation. Adopting an expectancy-value perspective, we set up a structural equation (*N* = 167) and two linear regression models (*n*_1_ = 86, *n*_2_ = 81) to examine the effects of student teachers’ career choice motives on relational goal orientation. Analyses showed that the relational motive of educational interest was the only significant predictor in a structural equation model with educational interest, subject-specific interest, and general ability beliefs as predictors and relational goal orientation as the criterion. The first regression model found that the social utility motive work with children/adolescents was a significant predictor of relational goal orientation when combined with other career choice motives, namely educational interest, subject-specific interest, general abilitiy beliefs, and three other social utility factors. The second regression model found no significant effects of educational interest, subject-specific interest, educational self-concept and subject-specific self-concept on relational goal orientation. The results suggest that teachers who choose their profession because they enjoy working with children and adolescents are likely to strive to build satisfactory student-teacher relationships. Implications for future research and teacher education are discussed.

## 1. Introduction

Educating children and adolescents is at the core of the teaching profession (Stürmer and Gröschner, 2022), and building professional student-teacher relationships is an integral part of the professional profile of teachers. Not surprisingly, the desire to work with children and adolescents is one of the most important reasons for choosing teaching as a career (Fox, 1961; Joseph and Green, 1986; Oesterreich, 1987; Eberle and Pollak, 2006; Watt and Richardson, 2008; Pohlmann and Möller, 2010; Berger and D’Ascoli, 2012; Fokkens-Bruinsma and Canrinus, 2012; Kılınç et al., 2012; Lin et al., 2012; Retelsdorf and Möller, 2012). This relational value should, therefore, also influence the types of goals (student) teachers pursue in the classroom. Goal orientation theory serves as a framework to describe what (student) teachers might intend to do when they teach (Butler, 2007; Dickhäuser et al., 2007; Nitsche et al., 2011; Janke et al., 2015): they can focus on learning from their teaching experience (learning goal orientation or mastery), demonstrating their teaching abilities (achievement approach), avoiding failure (achievement avoidance), avoiding workload (work avoidance), and building satisfactory relationships with their students (relational). Learning goal orientation and relational goal orientation have positive effects on student teachers’ professional development and their students’ learning (Dickhäuser et al., 2007; Nitsche et al., 2013a,b; Butler and Shibaz, 2014). Reasons for teachers’ career choices, namely educational or subject-specific interest and ability beliefs, have been shown to correlate with mastery goal orientation (Pohlmann and Möller, 2010; Paulick et al., 2013). However, no studies have investigated relational reasons for career choice as predictors of relational goal orientation. This study aimed to determine which of the factors that influence student teachers’ career choice predict relational goal orientation before student teachers’ first teaching experience.

## 2. Theoretical background

### 2.1. Importance of relational values for the career choice of teachers

Recent approaches to describing student teachers’ reasons for choosing teaching as a career mostly build on the expectancy-value framework (Eccles and Wigfield, 1995; Wigfield and Eccles, 2000) and understand career choice as an outcome of an individual’s expectancies for success (e.g., their ability beliefs) and the values attached to the task of choice (e.g., the intrinsic interest in teaching; Watt and Richardson, 2007; Pohlmann and Möller, 2010). Expectancy components include ability beliefs. Value components include intrinsic values, such as educational and subject-specific interest; personal utility values, such as job security or time for family; and social utility values, such as the desire to work with children and adolescents. Ability beliefs describe an individual’s self-evaluation of their capabilities in relation to the teaching profession. Intrinsic values are concerned with the pleasure individuals experience when performing job-related tasks such as educating, teaching, or learning more about their subject. Personal utility values describe values relevant for an individual’s life aspirations. Social utility values describe values such as supporting others in their personal development or contributing to society.

In most studies, intrinsic or interest components, social utility values, and ability beliefs have emerged as relevant predictors of career choice; extrinsic factors such as job security or time for family (personal utility) were not as important (Watt and Richardson, 2007; Retelsdorf and Möller, 2012; Watt et al., 2012). Except for general ability beliefs and personal utility values, all reasons for career choice described by the expectancy-value framework include a specific relational component. For intrinsic values, researchers have found both educational interest and subject-specific interest to be of importance — the former indicating the pleasure student teachers experience when supporting individual students in their personal development (Pohlmann and Möller, 2010; Schiefele and Schaffner, 2015). For social utility values, Watt and Richardson (2007, 2008) have identified four value aspects that lead adolescents toward teaching careers: they choose to become teachers because they consider it important to shape the future of children and adolescents, enhance social equity, make a social contribution in general, or simply to work with children or adolescents. In this study, we subsumed educational interest and social utility values under the term relational values because they have in common the pleasure or importance student teachers attribute to getting involved with children and adolescents and student teachers’ willingness to work toward a satisfactory teacher-student relationship. The evidence that relational values are important for teachers’ career choice builds upon a large body of older, mostly exploratory studies investigating teachers’ reasons for career choice conducted worldwide (Brookhart and Freeman, 1992; Watt and Richardson, 2007; Richardson and Watt, 2014). Most of those studies identified the desire to work with children and adolescents as the dominant reason, followed by other intrinsic motives (Fox, 1961; Joseph and Green, 1986; Oesterreich, 1987; Eberle and Pollak, 2006). Research within the expectancy-value framework has a similar conclusion. In Australia, highly engaged teachers show, in addition to high intrinsic motivation, high social utility values (Watt and Richardson, 2008). Preservice teachers from China, Turkey, and the United States all report that social utility values are among the most important reasons for career choice (Kılınç et al., 2012; Lin et al., 2012). In Switzerland and the Netherlands, social utility values were not the most important factor, but, in addition to perceived abilities and intrinsic motivation, were significant for career choice and affective commitment (Berger and D’Ascoli, 2012; Fokkens-Bruinsma and Canrinus, 2012). In Germany, the importance of educational interest and subject-specific interest has been reported (Pohlmann and Möller, 2010; Retelsdorf and Möller, 2012).

Both relational values and general ability beliefs are relevant predictors of career choice and persistence (Watt and Richardson, 2007; Retelsdorf and Möller, 2012; Watt et al., 2012). When student teachers evaluate their abilities regarding the teaching profession, they may also consider relational aspects, such as their skills in building relationships with students. Retelsdorf et al. (2014) presented evidence for the existence of six facets of (student) teachers’ professional self-concept, describing ability beliefs about subject-specific skills, educational skills, diagnostic skills, skills concerned with the innovation of teaching and school life, skills in handling digital media, and counseling skills. According to our review of the literature, no studies have considered those differing ability beliefs as separate predictors of career choice. Distinguishing career choice motivation from motivation within the teaching profession might be important. Student teachers can either evaluate the extent to which values and ability beliefs were and are important reasons for their decision to become a teacher or undertake self-evaluations of their abilities or interest statements in general. Studies of facets of student teachers’ ability beliefs (Retelsdorf et al., 2014) or interest (Schiefele et al., 2013) have not been concerned with career choice motivation. Therefore, whether educational and subject-specific self-concepts have different effects on student teachers’ career choice is unknown. This study assumes that, especially early in teacher education, when student teachers have little teaching experience, career choice motivation is salient and, therefore, highly influential (e.g., when student teachers enter the classroom for the first time). Relational values, shown to be important in career choice, could then play a critical role in determining student teachers’ intentions and goals.

### 2.2. Relational goal orientation of (student) teachers

Goals have been defined as cognitive representations of competence-related end states that individuals want to either approach or avoid and that guide their behavior (see also definition by Hulleman et al., 2010, page 423). Goal orientations describe an individual’s disposition to habitually strive for a certain type of goal in learning and achievement situations. Researchers originally distinguished ego-involved and task-involved goals (Nicholls, 1989). Ego involvement describes a disposition to strive for positive evaluations by others in achievement situations; task involvement describes the disposition to strive for mastery of the task at hand. Similarly, performance can be separated from learning or mastery goals (Dweck, 1986). Performance goals can further be divided into performance-approach goals (i.e., striving to show one’s competence) and performance avoidance goals (i.e., striving to avoid the appearance of failure). In addition to goal orientations concerned with the ego or the task, Wentzel (1994) and Urdan and Maehr (1995) have described goal orientations concerned with social relationships. By adapting the framework of mastery, performance-approach, and performance avoidance, social goal orientations can be divided into social development (striving for the development of social competence, e.g., by developing good friendships), social demonstration approach (striving for the demonstration of social competence, e.g., by being socially accepted), and social demonstration-avoidance (striving not to appear to be socially incompetent) goal orientations (Ryan and Shim, 2006; Jeanne Horst et al., 2007). Subsequently, Levontin and Bardi (2018, 2019) added the so-called amity goal orientation, a tendency to support others in their learning and achievement. Typically, learning or mastery goals are associated with adaptive behavior in achievement situations, whereas performance avoidance goals tend to have negative outcomes (Hulleman et al., 2010). The same pattern emerges for the social goal orientations of students: even if social goal orientations do not affect performance, they seem to be related to peer acceptance and well-being (Wentzel, 1994). By contrast, social development goal orientation is associated with positive emotions and adaptive coping, and social demonstration-avoidance goal orientation is linked to relationships with negative emotions such as fear, shame, or sadness (Shim et al., 2013; Michou et al., 2016). Only amity goal orientation has been shown to also affect performance when combined with mastery goals (Levontin and Bardi, 2018, 2019).

Butler (2007) and Dickhäuser et al. (2007) adapted the goal orientation approach for (student) teachers, with similar results. For teachers, learning goal orientation focuses on learning from experiences as a teacher to develop competence. Performance goal orientation is concerned with demonstrating teaching abilities or not showing incapability or problems with teaching. Work avoidance reflects teachers’ tendency to attempt to reduce their workload. Mostly, teachers’ achievement goals are concerned with teaching competence, including all aspects of the teaching process (Dickhäuser et al., 2007). Learning goal orientation has positive effects on instruction (Retelsdorf and Günther, 2011; Butler and Shibaz, 2014) and is associated with adaptive behavior related to student teachers’ professional development, such as asking for help (Dickhäuser et al., 2007) and pursuing further education (Nitsche et al., 2013a,b). Stating that teaching is a personal and interpersonal endeavor, Butler (2012) added relational goal orientation to the framework of teachers’ goals. She stated that for teachers, developing a satisfactory, professional relationship with their students was an important part of their professional profile. In the teaching context, relational goal orientation describes this focus on the development of satisfactory student-teacher relationships. Unsurprisingly, relational goal orientation in teachers has been associated with beneficial outcomes for student learning, namely social support, mastery learning, and student help seeking (Butler, 2012; Butler and Shibaz, 2014).

### 2.3. Relational values and relational goal orientation

Teachers’ reasons for choosing their careers are associated with their goal orientations (Pohlmann and Möller, 2010; Paulick et al., 2013). Interest and ability beliefs, for example, are related to task involvement, whereas personal utility values are correlated with ego involvement (Pohlmann and Möller, 2010). Paulick et al. (2013) found a similar pattern: mastery and performance-approach goal orientation is associated with both interest and ability beliefs, and performance avoidance and work avoidance goal orientation is associated with personal utility values. Intrinsic values and ability beliefs, therefore, seem to affect cognitive representations guiding teaching behavior associated with the development of competence as a teacher, with beneficial outcomes for instruction (see also Paulick et al., 2013). None of the studies included relational goal orientation. We assume that particulary relational values for choosing a career (educational interest and social utility values) predict setting goals concerned with developing a satisfactory student-teacher relationship. General ability beliefs concerning the profession may also play a role when student teachers think of relational skills as they evaluate their teaching abilities. There is a positive association between ability beliefs and learning goal orientation (Pohlmann and Möller, 2010; Paulick et al., 2013) that might also be true for relational goal orientation, owing to both orientations sharing a focus on development that becomes evident in their empirical correlation (Butler, 2012; Butler and Shibaz, 2014; Daumiller et al., 2019). However, beliefs about abilities concerning building relationships can only be one part of general ability beliefs, with subject-specific and didactical skills also being important aspects of the profession. Furthermore, expectancy-value theory classifies goal orientations as being conceptually closer to the value component than to the expectancy component (Eccles and Wigfield, 2002), suggesting that especially interest and value should be associated with goal orientations.

### 2.4. Research questions and hypotheses

The objective of this study was to investigate the association between relational factors of career choice motivation and relational goal orientation to fill the research gap regarding predictors of relational goal orientation at the early stages of student teachers’ professional development. Educational interest and social utility values should predict relational goal orientation better than other reasons for choosing a teaching career, namely subject-specific interest and general ability beliefs. We hypothesized that educational interest is a better predictor of relational goal orientation than subject-specific interest and general ability beliefs (Hypothesis 1). The second step was to analyze the effects of social utility values. Social utility values were expected to explain additional variance in relational goal orientation, adding to the effects of educational interest (Hypothesis 2). Because general ability beliefs concerning the teaching career may include both relational and other aspects of the profession, we attempted to separate those components in a third analysis to assess whether educational ability beliefs, in addition to relational values, affect relational goal orientation. Therefore, this study examined the combined effects of educational interest, subject-specific interest, educational self-concept, and subject-specific self-concept. Hypothesis three was that among those four factors, only educational interest predicts relational goal orientation.

## 3. Materials and methods

### 3.1. Participants and procedure

With regard to the planned analyses, a sample of approximately 200 student teachers was aimed for based on the suggestions of Wolf et al. (2013). For this purpose, it was planned to recruit student teachers in a bachelor’s degree program preparing for a career as a teacher at a German Gymnasium in a compulsory course prior to their first teaching practicum over the course of two terms, since approximately 100 people participated in this study element per term. By recruiting participants in a course immediately prior to their first practicum, we wanted to survey them at a time when increased interest in the survey could be expected.

The final sample combined the two samples from two cohorts of student teachers in a bachelor’s degree program preparing for a career as a teacher at a German Gymnasium at a large German university. Participants were recruited in two consecutive terms (winter term 2016 and summer term 2017) in a mandatory preparation course for their three-week orientation practicum, their first teaching practicum in the degree program. This internship is designed to help student teachers ascertain their career choice, so we assumed that career choice motivation would be particularly salient at this time. Student teachers enrolled in the courses were contacted *via* email and asked to participate; they were then sent a link to an online questionnaire. Surveys were administered prior to the start of the courses and participation was voluntary. Voluntary participation was one of the measures used to prevent Careless Responding (CR), which is particularly common in online surveys; participants were also asked to respond honestly and conscientiously (for details about prevention of CR in survey data, see Ward and Meade, 2023). A total of *N* = 227 student teachers were enrolled in the course in the two terms. *N* = 86 questionnaires were completed in the winter term 2016 (sample 1), and *N* = 97 questionnaires were completed in the summer term 2017 (sample 2). From the questionnaires completed in the summer term 2017, we excluded 15 cases because not all scales were assessed in these due to an error in the survey settings; a further case was excluded because there was evidence of careless responding, indicated by a large amount of consecutive identical responses.

This study conducted analyses with samples 1 and 2 as stand-alone samples, as well as using both samples together (hereafter the combined sample) as a data set (see also section on data analysis). The combined sample comprised 167 student teachers (113 female, 54 male): *n*_1_ = 86 (60 female, 26 male) and *n*_2_ = 81 (53 female, 28 male); and *M*(age) = 20.66, *SD*(age) = 1.71; *M*_1_(age) = 20.83, *SD*_1_(age) = 1.64; *M*_2_(age) = 20.48, and *SD*_2_(age) = 1.78. The samples did not differ in their reasons for choosing teaching as a career (*p* > 0.05 for educational interest, subject-specific interest, and general ability beliefs) or their prior educational experience (*p* > 0.05), but relational goal orientation was higher in sample 2 than in sample 1 (*p* < 0.01, *M*_1_ = 3.99, *SD*_1_ = 0.54, *M*_2_ = 4.26, *SD*_2_ = 0.63). With *N* = 167 participants, the combined sample comprised 73% of the student teachers enrolled in the respective courses.

### 3.2. Measures

#### 3.2.1. Combined sample

For organizational reasons, not all measures could be collected at both measurement times. The scales that were part of both assessments are presented first. Subsequently, a description of the scales for the respective sample is presented (see overview in Table 1), including information concerning validity and reliability estimates. This study calculated Cronbach’s alpha as a widely-known estimator of internal consistency, with the understanding that it should be approached with caution (Sijtsma, 2009). Therefore, measurement models using confirmatory factor analysis (CFA) were included in the analyses to gain a better understanding of the scales.

**Table 1:** Overview of all study variables and their collection in the respective samples.

Study variables	Samples		
	Combined sample	Sample 1	Sample 2
*Sociodemographic and control variables*	+		
Age	+		
GPA Abitur	+		
Gender	+		
Prior pedagogical learning gain	+		
*Reasons for choosing teaching as a career* (*FEMOLA*, Pohlmann and Möller, 2010)	+		
Educational interest	+		
Subject-specific interest	+		
General ability beliefs	+		
*Relational goal orientation* (Daumiller et al., 2016)	+	+	+
*Social utility values for choosing teaching as a career* (*FIT-Choice*, Watt et al., 2012)		+	
Shape future of children/adolescents		+	
Enhance social equity		+	
Make social contribution		+	
Work with children/adolescents		+	
*Specific self-concepts* (*ERBSE-L*, Retelsdorf et al., 2014)			+
Subject-specific self-concept			+
Educational self-concept			+

##### 3.2.1.1. Sociodemographic variables

Participants indicated their age (*M* = 20.66, *SD* = 1.71), their gender (113 female, 54 male), and the grade point average of their *Abitur* (German high school degree qualifying for university studies; *M* = 2.01, *SD* = 0.58, with 1 indicating the best and 6 indicating the worst grade) before filling in the questionnaire.

##### 3.2.1.2. Reasons for choosing teaching as a career

This study used the German Motivation for Choosing Teacher Education Questionnaire, FEMOLA (Pohlmann and Möller, 2010) to assess the expectancy and value components of student teachers’ reasons for choosing teaching as a career. The questionnaire was developed by Pohlmann and Möller (2010), whose original study on the development of the scale demonstrated validity by examining the factor structure and associations with other variables close to the FEMOLA constructs in three different samples of student teachers. This study’s sample is comparable to the samples used by Pohlmann and Möller (2010). Participants indicated why they had chosen to become a teacher on a 4-point Likert scale ranging from *does not apply at all* to *fully applies*. Value components were measured with the FEMOLA subscales for educational interest (*α* = 0.83 in the combined sample, *α_1_* = 0.82 in sample 1, *α_2_* = 0.83 in sample 2, item example: “I like to work with children and adolescents.”) and subject-specific interest (*α* = 0.65, *α_1_* = 0.58, *α_2_* = 0.69, item example: “…my subjects are important to me.”). The subscale ability beliefs (*α* = 0.77, *α_1_* = 0.82, item example: “…I think I can be a good teacher.”) assessed an expectancy component.

##### 3.2.1.3. Relational goal orientation

The four German items to assess relational goal orientation were developed by Daumiller et al. (2016) for university instructors and adapted for student teachers by changing the German term for university students to school students. Participants indicated on a 5-point Likert scale (ranging from *not at all true* to *totally true*) which goals they aimed for in their profession as a teacher (*α* = 0.61, *α_1_* = 0.58, *α_2_* = 0.73), item example: “…it is important to me to achieve a personal connection with my students.”). In their original study, Daumiller et al. (2016) provided evidence for the factor structure of their goal orientation scales and for associations with other constructs in a sample of university instructors, following up on the studies of Butler (2012) and Butler and Shibaz (2014) who validated the relational goal orientation scale in samples of teachers.

#### 3.2.2. Additional measures in sample 1: Social utility values

This study used a German version of the Factors Influencing Teaching Choice (FIT-Choice) scale (Watt et al., 2012) to assess additional reasons for choosing teaching as a career. Participants indicated which reasons were important to their decision to become a teacher on a 7-point Likert scale (ranging from *not important at all* to *very important*). The items that assess social utility values can be aggregated into one social utility higher order factor; another possibility is to calculate four different social utility sub-factors, allowing for a detailed analysis of value components that might be associated with relational goal orientation (Watt and Richardson, 2007). The FIT-Choice scale has been used in samples of teachers and student teachers in many studies around the world (Watt et al., 2012; Watt and Richardson, 2012). Item examples are “Teaching will allow me to influence the next generation” for the sub-factor *shape future of children/adolescents* (*r* = 0.46), “Teaching will allow me to benefit the socially disadvantaged” for *enhance social equity* (*r* = 0.67), “Teaching makes a worthwhile social contribution” for *make a social contribution* (*α* = 0.72), and “I want to work in a child/adolescent-centered environment” for *work with children/adolescents* (*α* = 0.89).

#### 3.2.3. Additional measures in sample 2: Specific self-concepts

In addition to the general ability beliefs factor assessed with the FEMOLA scales, this study included a fine-grained measure of professional self-concepts for teaching, the ERBSE-L (*Erfassung berufsbezogener Selbstkonzepte von angehenden Lehrkräften*) scales (Retelsdorf et al., 2014). The ERBSE-L scales include, among others, subscales for subject-specific (*α* = 0.79) and educational (*α* = 0.68) self-concepts of (student) teachers (4-point Likert scale, where participants indicate whether a statement applies to them, ranging from *does not apply at all* to *fully applies*). Notably, self-concept is not considered a reason for a career choice in this instrument but is part of student teachers’ professional motivations.

#### 3.2.4. Control variables in all samples

To determine the prior teaching or educational experience of the participants, they were asked how much they had learned in previous internships or on similar occasions with regard to their educational skills (one item format). They estimated their prior educational learning gain on a 7-point Likert scale ranging from *very low* to *very high*. In addition to educational experience, we considered gender a potential control variable because it has been associated with career choice motivations in the literature (Ulich, 1998; Retelsdorf et al., 2010). Furthermore, women tend to report higher learning goal orientation (Butler, 2007, 2012), educational interest (Schiefele and Schaffner, 2015), and educational self-concept (Retelsdorf et al., 2014) than men do.

### 3.3. Data analysis

This study conducted preliminary analyses and analyzed bivariate correlations of all study variables for the combined sample, sample 1 and sample 2 to check the assumptions for the analyses. To test the first hypothesis, we set up a structural equation model using the R package lavaan (Rosseel, 2012) with data from the combined sample. Before testing hypothesis 1, we tested measurement models for the scales measuring interest (educational and subject-specific) and ability beliefs as reasons for choosing teaching as a career, and especially for the adapted scale measuring relational goal orientation of student teachers. We then assessed a structural model with direct effects of the career choice factors educational interest, subject-specific interest, and general ability beliefs on relational goal orientation while controlling for the inter-correlations of the three predictors and effects of prior pedagogical learning gain. All included constructs were modeled as latent factors. Model fit was evaluated *via* absolute fit indices (root mean square error of approximation, RMSEA; comparative fit index, CFI; standardized root mean square residual, SRMR), considering the suggestions by Schermelleh-Engel et al. (2003). All models were investigated using the maximum likelihood estimator (ML). Hypotheses 2 and 3 were investigated with data from samples 1 and 2, respectively. Due to the relatively small sample size, this study used linear regression analysis instead of structural equation modeling. To test the second hypothesis, we regressed relational goal orientation on the four social utility factors and the same variables as in the structural equation model (educational interest, subject-specific interest, and general ability beliefs). To test the third hypothesis, we set up a regression model with the same interest factors as used in the structural equation model (educational interest, subject-specific interest) but added specific ability belief components: the variables educational self-concept and subject-specific self-concept. Notably, the self-concept variables were not assessed as reasons for career choice, as all other predictors were, but as self-assessments of abilities in the profession. Prior pedagogical learning gain and gender were control variables in both regression models.

## 4. Results

### 4.1. Preliminary analyses

#### 4.1.1. Univariate normality

All scales in the combined sample and sample 1 and sample 2 significantly deviated from univariate normality (*p* < 0.05 in all Shapiro-Wilks tests). This study, therefore, used robust methods if possible. The confirmatory factor analysis and structural equation models were estimated with the maximum likelihood estimator (ML), which is quite robust against the violation of the normality asumption. This estimator was chosen instead of a weighted least squares estimator, which should be considered when using scales with only four scale points as in this study, and instead of a robust maximum likelihood estimator because of the relatively small sample size (see suggestions by Schermelleh-Engel et al., 2003). For the regression analyses, we followed recommendations by Field and Wilcox (2017).

#### 4.1.2. Bivariate correlations

Table 2 presents correlations of all study variables. On a bivariate level, relational goal orientation was associated with educational interest in all samples (0.26 < *r* > 0.44, *p* < 0.05), general ability beliefs in the combined sample (*r* = 0.21, *p* < 0.05), all four social utility values in sample 1 (0.28 < *r* > 0.46, *p* < 0.01), and subject-specific interest (*r* = 0.23, *p* < 0.05) and educational self-concept (*r* = 0.26, *p* < 0.05) in sample 2. None of the correlations indicated problems in performing regression analyses, with one exception. Educational interest and the social utility factor *work with children/adolescents* were highly correlated (*r* = 0.73, *p* < 0.001, sample 1), indicating that distinguishing the two constructs was difficult in this study.

**Table 2:** Descriptive values and bivariate correlations of all study variables.

	*M*	*SD*	1	2	3	4	5	6	7	8	9	10
*Combined sample*												
1 Relational goal orientation	4.12	0.61										
2 Educational interest	3.52	0.42	0.36^***^									
3 Subject-specific interest	3.43	0.50	0.13	0.29^***^								
4 General ability beliefs	3.29	0.46	0.21^**^	0.43^***^	0.20^*^							
5 Prior pedagogical learning gain	4.55	1.50	0.14	0.30^***^	−0.03	0.23^**^						
*Sample 1*												
1 Relational goal orientation	3.99	0.54										
2 Educational interest	3.48	0.45	0.44^***^									
3 Subject-specific interest	3.49	0.43	0.05	0.25^**^								
4 General ability beliefs	3.29	0.50	0.23	0.54^***^	0.22							
5 Prior pedagogical learning gain	4.57	1.40	0.10	0.22^*^	−0.01	0.20						
6 Shape future of children/adolescents	6.16	0.89	0.35^**^	0.60^***^	0.28^*^	0.49^***^	0.01					
7 Enhance social equity	5.61	1.11	0.46^***^	0.46^***^	0.30^*^	0.28^**^	0.14	0.56^***^				
8 Make social contribution	5.89	1.00	0.30^**^	0.37^***^	0.19^*^	0.31^**^	0.15	0.51^***^	0.65^***^			
9 Work with children/adolescents	5.76	1.12	0.28^***^	0.73^***^	0.21^*^	0.32^**^	0.21^*^	0.32^**^	0.33^**^	0.19^*^		
*Sample 2*												
1 Relational goal orientation	4.26	0.63										
2 Educational interest	3.57	0.39	0.26^*^									
3 Subject-specific interest	3.37	0.55	0.23^*^	0.35^**^								
5 Prior pedagogical learning gain	4.53	1.60	0.17	0.40^***^								
10 Educational self-concept	3.24	0.46	0.23^*^	0.50^***^	0.26^*^		0.43^***^					
11 Subject-specific self-concept	3.13	0.51	−0.01	−0.06	0.27^*^		−0.15					0.04

^*^p < 0.05, ^**^p < 0.01, ^***^p < 0.001.

#### 4.1.3. Missing values

In sample 1, there were two missings on prior pedagogical learning gain and one missing on one item measuring subject-specific interest. In sample 2, there was one missing on prior pedagogical learning gain. Consequently, in the combined sample, there were three missings on prior pedagogical learning gain and one missing on one indicator measuring subject-specific interest. For the CFA and SEM models, we used the full information maximum likelihood (FIML) method to estimate the models including all cases. In the regression analyses, we opted for listwise deletion because the amount of missing data was very small (< 2% for all variables in sample 1 and < 1% for all variables in sample 2).

### 4.2. Effects of educational interest on relational goal orientation (Hypothesis 1)

#### 4.2.1. Factor structure

We set up a confirmatory factor analysis (CFA) in R and evaluated model fit of measurement models for relational goal orientation, and for interest and ability beliefs as reasons for career choice *via* absolute fit indices (root mean square error of approximation, RMSEA; comparative fit index, CFI; standardized root mean square residual, SRMR). The first model tested was one with a common factor for all interest items (seven items for educational interest and four items for subject-specific interest), a common factor for all items measuring ability beliefs and a common factor for all items measuring relational goal orientation (Model 1). In a second model, the items for educational interest and the items for subject-specific interest loaded on separate interest factors (Model 2), as recommended by Pohlmann and Möller (2010). In a third alternative model one of the items assessing relational goal orientation was excluded because it had shown a low loading in the previous two models and also differed in content from the other three items measuring this factor (Model 3). Table 3 shows fit indices of all three models. Model fit was sufficient for Model 3, indicating that educational interest and subject-specific interest should be considered as separate factors and relational goal orientation should be estimated on the basis of three items instead of the four items of the original scale in the following analysis. The model then was extended by regressing relational goal orientation on the two interest factors and the ability beliefs factor in a first step (Model 4), and by adding prior pedagogical learning gain as control variable in a second step (Model 5). Fit indices of all models are depicted in Table 3.

**Table 3:** Model Fit of the measurement models und structural models.

Model	*ꭓ*^2^	df	CFI	RMSEA+	SRMR	AIC	BIC
Model 1	392.73	167	0.770	0.090 [0.078, 0.102]	0.090	6198.29	6394.73
Model 2	318.75	164	0.842	0.075 [0.063, 0.087]	0.080	6130.31	6336.10
Model 3	267.82	146	0.870	0.071 [0.057, 0.084]	0.080	5702.85	5899.28
Model 4	267.82	146	0.870	0.071 [0.057, 0.084]	0.080	5702.85	5899.28
Model 5	289.33	163	0.869	0.068 [0.055, 0.081]	0.079	6287.04	6495.94

df, degrees of freedom. CFI, comparative fit index. RMSEA, root mean square error of approximation. SRMR, standardized root mean square residual. AIC, Akaike Information Criterion. BIC, Bayesian Information Criterion. +: 90% confidence interval in brackets.

#### 4.2.2. Predicting relational goal orientation from reasons for career choice

**Figure 1:**
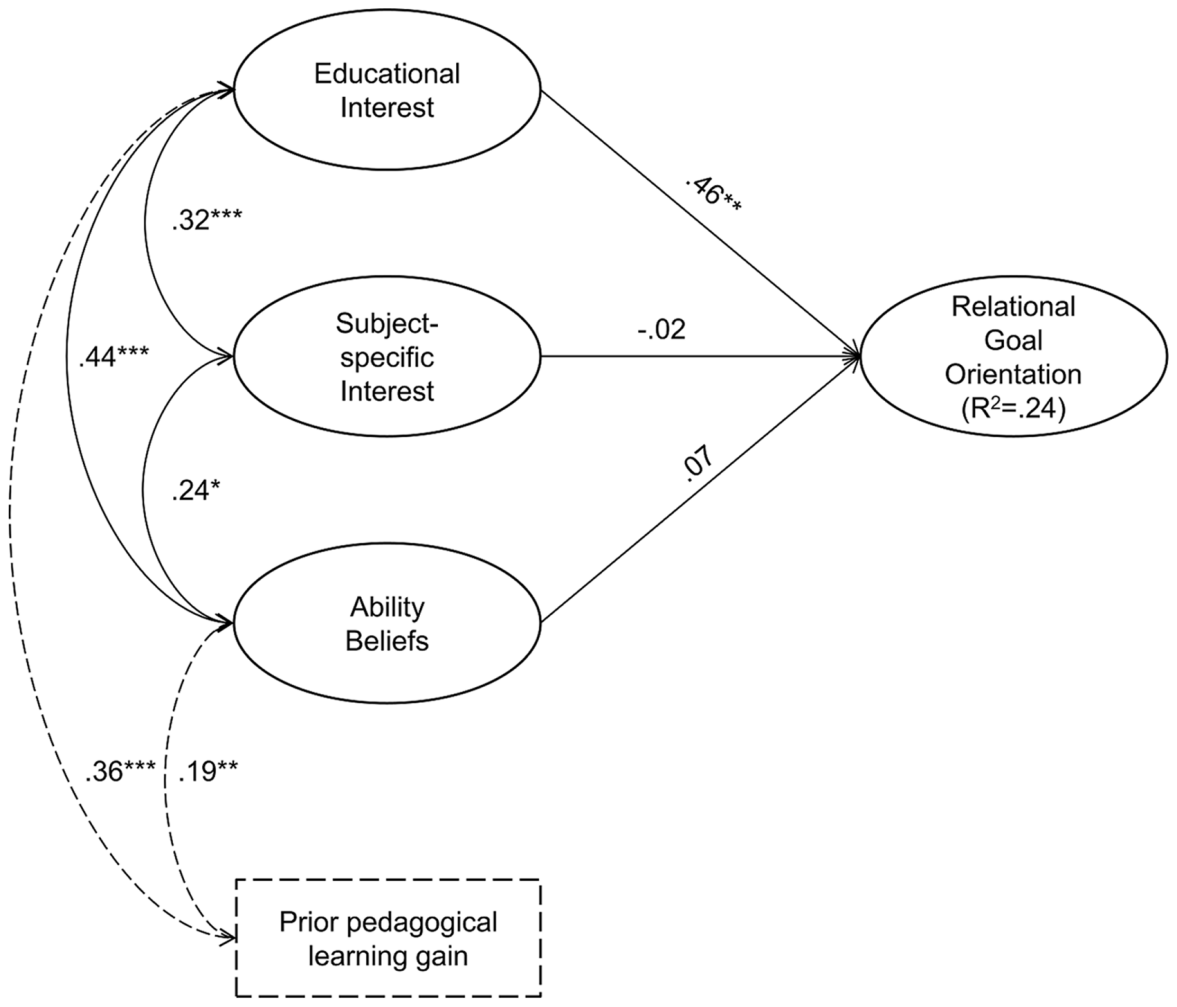
Model 5 depicting the relationships among relational goal orientation, educational interest, subject-specific interest and general ability beliefs.

Figure 1 shows the model results of the final structural equation model with educational interest, subject-specific interest, and general ability beliefs as predictors and relational goal orientation as the criterion, and with prior pedagogical learning gain as a control variable (*N* = 167). All intrinsic motivation latent factors were allowed to correlate. Model fit was sufficient (Model 5: *χ*^2^(163) = 289.33, *p* < 0.001, *SRMR* = 0.08, *RMSEA* = 0.07, *CFI* = 0.87). Model results support hypothesis 1, with educational interest emerging as the only relevant predictor of relational goal orientation in the model.

### 4.3. Effects of social utility values on relational goal orientation (hypothesis 2)

In the robust regression model with relational goal orientation as the criterion in sample 1 predictors were entered stepwise (see Table 4 for model results). Step 1 introduced the control variables prior pedagogical learning gain and gender. This study then replicated the results from the structural equation model in step 2, with educational interest being a significant predictor of relational goal orientation when entered with subject-specific interest and general ability beliefs (*R^2^* = 0.20). When the four social utility factors (*shape future of children/adolescents*, *enhance social equity*, *make social contribution*, and *work with children/adolescents*) were added in step 3, the effect of educational interest vanished. In the final model that explained 28% of the variance in goal orientation, only the social utility factor *work with children/adolescents* reached significance, partly supporting hypothesis 2. Educational interest and the factor *work with children/adolescents* were highly correlated in sample 1 (*r* = 0.73, *p* < 0.05).

**Table 4:** Contributions of social utility values to relational goal orientation.

	adjusted *ΔR*^2^	*B*	*SE*
Step 1	0.04		
Gender^a^		0.20	0.16
Prior pedagogical learning gain		0.04	0.14
Step 2	0.16		
Gender		0.26*	0.12
Prior pedagogical learning gain		0.01	0.03
Educational interest		0.69***	0.14
Subject-specific interest		0.00	0.13
General ability beliefs		−0.13	0.15
Step 3	0.08		
Gender		0.27*	0.12
Prior pedagogical learning gain		−0.01	0.03
Educational interest		0.25	0.27
Subject-specific interest		−0.12	0.13
General ability beliefs		−0.08	0.15
Shape future of children/adolescents		0.01	0.07
Enhance social equity		0.15°	0.08
Make social contribution		0.02	0.08
Work with children/adolescents		0.13*	0.06

*N* = 83.

°*p* < 0.10, *p < 0.05, **p < 0.01, ***p < 0.001.

a Scoring of gender: male = 1, female = 2.

### 4.4. Effects of specific ability beliefs on relational goal orientation (hypothesis 3)

A robust regression model with sample 2 with relational goal orientation as criterion introduced the control variables prior pedagogical learning gain and gender in step 1. In step 2, this study used the same predictors as in the structural equation model and replaced the predictor general ability beliefs with specific factors: educational self-concept and subject-specific self-concept. Hypothesis 3 was not supported because there were no significant effects of any predictors on relational goal orientation (see Table 5 for model results).

**Table 5:** Contributions of specific self-concepts to relational goal orientation.

	adjusted Δ*R*^2^	*B*	*SE*
Step 1	0.11		
Gender^a^		−0.32*	0.23
Prior pedagogical learning gain		0.07°	0.15
Step 2	0.04		
Gender		−0.24	0.16
Prior pedagogical learning gain		0.06	0.05
Educational interest		0.23	0.22
Subject-specific interest		0.13	0.17
Educational self-concept		−0.01	0.22
Subject-specific self-concept		0.08	0.16

*N* = 80.

°*p* < 0.10, ^*^p < 0.05, ^**^p < 0.01, ^***^p < 0.001.

a Scoring of gender: male = 1, female = 2.

## 5. Discussion

The results of this study support the hypothesis that individuals who become teachers because they value working with children and adolescents intend to follow relational goals in their working environment. This study fits well with other studies on the relationship between career choice motivation and goal orientations that have shown an association between the interest and ability components of career choice motivation and mastery goal orientation (Pohlmann and Möller, 2010; Paulick et al., 2013). The study results fill the research gap regarding the association of the relational components of both constructs. This study found evidence for an association between relational values (educational interest and social utility values) and relational goal orientation. As hypothesized, educational interest had a substantial effect on relational goal orientation (Hypothesis 1). Whereas educational and subject-specific interests have emerged as predictors of learning goal orientation (Paulick et al., 2013), for relational goal orientation, only relational values affecting career choice were of importance in this study. The social utility value *work with children/adolescents* also predicted relational goal orientation (Hypothesis 2), but the effects were not clearly separable from the effects of educational interest. The high correlation of educational interest with the factor *work with children/adolescents* in the regression analysis with sample 1 (*r* = 0.73, *p* < 0.001) hints at problems with multicollinearity and makes interpreting the effects of both predictors separately difficult. Furthermore, this strong correlation indicates that educational interest and social utility values share a component that incorporates student teachers’ pleasure in working with children and adolescents. When this component is an important reason to become a teacher, student teachers are likely to set relational goals when entering the classroom for teaching. This study did not find an effect of general ability beliefs on relational goal orientation, but, in line with Paulick et al. (2013), these beliefs were associated on a bivariate level (*r* = 0.21, *p* < 0.01). When evaluating their abilities for the teaching profession with items such as “Teaching is a career suited to my abilities,” student teachers might also consider relationship-related aspects of the profession. To improve the understanding of the effects of relationship-related aspects of ability beliefs, specifically assessing educational ability beliefs as part of student teachers’ choice motivations is necessary. According to our review of the literature, there are no such scales; therefore, this study assessed educational and subject-specific self-concepts within the profession in sample 2. Hypothesis 3 was not confirmed because none of the four components were significant predictors of relational goal orientation in this study. Consequently, the effects of educational interest from the structural equation model with the combined sample were not replicated in sample 2 either. Because the values for relational goal orientation were especially high in sample 2, ceiling effects may have limited the variance of the criterion variable. Missing effects could then have statistical causes, although this study used robust methods. Therefore, evaluating the missing effects of educational interest and educational self-concept on relational goal orientation in the regression model with sample 2 is difficult. Considering all analyses, we could also conclude that the reasons for career choice showed the expected effects, but self-concepts within the profession did not. Because the participants in this study had not yet gained practical experience, they may have been at an early stage in developing their self-concept of the vocational field. Therefore, a valid assessment of specific components of their self-concepts within the profession might be difficult. The reasons for career choice, on the other hand, were likely the result of in-depth exploration before entering a teaching training program. The fact that the student teachers in this study may have been more concerned with the reasons for career choice than with their professional self-concepts could be one explanation for the undetectable relationship between educational self-concept and relational goal orientation in sample 2.

### 5.1. Limitations

When interpreting the results, several limitations should be considered. First, the cross-sectional nature of the data did not allow causal interpretation of the empirical associations. Relational values could be the origin of relational goals; another possibility is that individuals who generally tend to set relational goals have many positive interactions and start to increasingly value social interaction and relationships. Therefore, prospective studies are necessary to improve the understanding of causal mechanisms or reciprocal influences. Second, the CFAs hinted at problems with one of the indicators measuring relational goal orientation. Therefore, this indicator was excluded from the structural equation model we used to investigate hypothesis 1. In this model, relational goal orientation was estimated from the items “…it is important to me to achieve a personal connection with students.,” “…it is one of my objectives to establish a partner-like relationship with students,” and “…it is my main objective to establish a positive relationship with my students,” but without the item “…I want to signal to my students that I have a genuine interest in their opinions and perceptions.” This study assumed that the scale measured student teachers’ cognitive representations guiding behavior toward the development of a positive relationship with their students. Before the scale is used in future studies, thorough validation is needed to improve understanding of the construct measured by the scale and to address reliability issues. Such studies could also investigate whether an assessment *via* a 5-point Likert-scale is appropriate to obtain an interval-scaled variable or whether the scale should be expanded to include additional scale points. The latter is also true for the FEMOLA and ERBSE-L scales with four scale points, which were treated as interval scales in this study and in previous studies. Negative skewness of intrinsic motivations also hinted at ceiling effects. This study attempted to minimize these effects using robust methods, but the ceiling effects indicate that there were mainly highly motivated student teachers in the study samples. Only 73% of the 227 student teachers in the two cohorts participated in this study, and because participation was voluntary, it is possible that the participants who completed the questionnaires were more motivated or interested than the 23% who did not participate. The generalizability of the results is, therefore, limited. Furthermore, the participants were at the beginning of their university studies and had not completed the practical elements of their teacher education program. The association between relational choice motivations and relational goal orientation might remain stable (see the results of Paulick et al., 2013, among practicing teachers), but the analyses of this study do not allow any conclusions to be drawn in this regard. This study can only make statements about highly motivated student teachers without practical educational experience. Because the scales assessing reasons for career choice (FEMOLA and FIT-Choice) include only a very general measurement of ability beliefs, and because those beliefs were correlated with relational values, evaluating the role of ability beliefs in relational goal setting is also difficult. Thus, further research is necessary that considers the educational and subject-specific aspects of ability beliefs as reasons for career choice.

### 5.2. Implications and future directions

Because of the importance of reasons such as the desire to work with children in choosing a teaching career and the association of those reasons with relational goal orientation, further research should consider relational goal orientation in addition to other goal orientations. Further research should also aim for a thorough validation and further development of the relational goal orientation scale. Relational goals could also be crucial for student teachers’ first experiences with classroom situations. In teacher education worldwide, practical experiences are part of the study program (Gröschner et al., 2015). During such teaching practicums, student teachers are asked to examine their motivations for choosing a career and to determine whether they want to pursue a teaching career and believe they are capable of doing so. Because choice motivations can be very important during this time, university courses or mentoring approaches that accompany internships should also discuss relational aspects of the teaching experience to include educational and social utility values (see also the proposal by Watt et al., 2014). In this context, further research could also examine the role of the approach and avoidance components of relational goal orientation. The scale used in this study measures a developmental component, similar to social developmental goal orientation in students (Shim et al., 2013; Michou et al., 2016). When teaching for the first time, student teachers may also be preoccupied with hiding the difficulties they may have in establishing a satisfactory working relationship with their students, reflecting more of a social demonstration/avoidance orientation. In addition, differences among relational goal orientation, social developmental goal orientation (see Michou et al., 2016), and pedagogical learning goal orientation (see Nitsche et al., 2011) may be of interest. Theoretically, relational goal orientation is about developing a relationship, social development goal orientation is about developing relationship competence, and educational goal orientation is about developing educational competence. Hulleman et al. (2010) defined a performance goal as “a future-oriented cognitive representation that directs behavior toward a competence-related end state that the individual either wants to approach or avoid” (see Hulleman et al., 2010, p. 423). Social development and educational goals fall under this definition, but relational goals can be considered to direct behavior toward a relational end state rather than a competency-related end state. In professions such as teaching, where there is always some type of interpersonal interaction, approach and avoidance tendencies toward relational end states might explain teachers’ experiences, behaviors, and success in the classroom, particularly because relational values are important in the decision to become a teacher.

## 6. Conclusion

This study provides new evidence on the effects of relational values in teachers’ career choice on relational goal orientation within the teaching profession, complementing other studies that have examined the relationship between reasons for career choice and goal orientations but did not consider the relational component. Because this study is an initial exploration of the topic, it has outlined important research questions. The findings also encourage reflection on the relational aspects of the teaching experience in courses that accompany student teachers’ initial field experiences.

## Data availability statement

The raw data supporting the conclusions of this article will be made available by the authors, without undue reservation.

## Ethics statement

Ethical review and approval was not required for the study on human participants in accordance with the local legislation and institutional requirements. The patients/participants provided their written informed consent to participate in this study.

## Author contributions

LO and SH conceptualized the study. LO collected the data, conducted data analysis, and wrote the first draft. SH supervised the project. All authors contributed to the article and approved the submitted version.

## Funding

This study was developed in a project supported by the German Ministry of Education and Research (BMBF), with funding provided by the ‘Qualitätsoffensive Lehrerbildung’.

For the publication fee we acknowledge financial support by Deutsche Forschungsgemeinschaft within the funding programme ‘Open Access Publikationskosten’ as well as by Heidelberg University.

## Conflict of interest

The authors declare that the research was conducted in the absence of any commercial or financial relationships that could be construed as a potential conflict of interest.

## Publisher’s note

All claims expressed in this article are solely those of the authors and do not necessarily represent those of their affiliated organizations, or those of the publisher, the editors and the reviewers. Any product that may be evaluated in this article, or claim that may be made by its manufacturer, is not guaranteed or endorsed by the publisher.
